# Safety and risk management of Chinese enterprises in Gabon's mining industry

**DOI:** 10.1016/j.heliyon.2023.e20721

**Published:** 2023-10-05

**Authors:** Ines Pamela Nguembi, Li Yang, Vincentia Serwah Appiah

**Affiliations:** School of Economics and Management, Anhui University of Science and Technology, Huainan, China

**Keywords:** Mining industry, Chinese enterprise, Mining risk, Safety management

## Abstract

The mining sector is booming due to the market and worldwide demand increase. The use of novel industrial processes and equipment, growth in trained personnel, and growth in managerial skills go hand in hand with the need to increase productivity in mining areas. These improvements make the mining industry one of the world's riskiest and most unstable. Several large-scale mining projects have failed due to a failure to recognize or underestimate risks, even when the industry uses risk management methods appropriately. To increase decision-making accuracy and protect mining organizations, risk mitigation of development work should be pursued. Safety and risk assessment is vital in the mining industry. The paper evaluates mining industry risks and safety. This research used Gabon's mining sector. This research included multiple dataset-gathering and interpretation methods. This study shows how to find a variety of recognized risks and uncertainty that have not been considered in any methodical or systemic approach to mining sector risk and safety monitoring. This research organizes risks hierarchically to show their consequences and propensity for each stage.

## 1 Introduction

The mining sector requires safety monitoring. The command-and-order system has reduced injury rates. A variety of methodological standards are now legally binding thanks to the Mining Acts and Guidelines. A thorough study is required to determine the possibility of disasters as a result of changing or worsening any of these measures. Modern mechanized mining requires significant financial outlay. These investments strive to increase revenue and well-being because the ecosystem threatens some assets. To attain the payback on the capital target, a yearly increase in productivity is required. Before spending more money, a mining corporation must assess its investment risks.

**Fig. 1:**
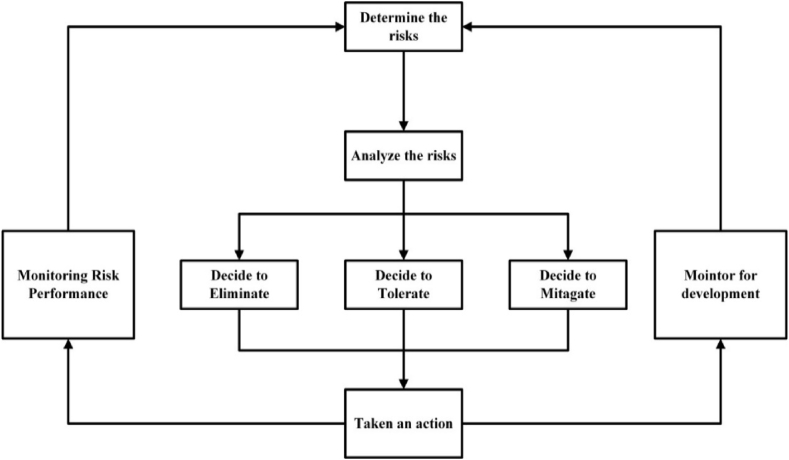
Framework for primary risk mitigation [1].

Risk evaluation examines threats that could harm a mining firm and its workers. According to the wide agreement, the risk evaluation method had a predetermined range to make workplaces safer. Risk refers to the likelihood and severity of injury. The mining industry involves employees, neighborhood ecology, company finances, and social well-being. Mining may seem innocent at first, but it may result in harm. Determining risky areas is key. That requires risk minimization. Fig. 1 shows the risk management structure [1].

Explorers, kings, merchants, governments, and enterprises have always sought Africa's minerals. As a result of the split of Africa by European imperial powers after the 19th century, Africans and their civilizations experienced terrible effects [2]. The southern African diamond and golden booms revealed Africa's natural resources, despite its lengthy history of mines and metallurgy. After emancipation and nationalization in the late nineteenth century, European and North American companies controlled African mines throughout the 20th century. Africa faced a new issue early in the 21st century. When indigenous assets weren't practicable, China's rapid rise in minerals demands and Africa's vast unknown riches lured Chinese foreign direct investment (FDI) into exploration and mining [3]. Between sensationalized and inflated assertions like “the Chinese seem eager to draw all possible strings, China's resources endeavor is worldwide and one of the greatest ambitious in existence,” to in-depth scientific work meticulously investigating various aspects of Chinese mining operation [4].

With the help of risk evaluation, mining personnel can identify severe, moderate, and low susceptibility. Risk evaluations can help optimize risk factors, provide more data on the likelihood of injury and its consequences, merge likelihood and seriousness evaluations to create a risk evaluation, and use that risk evaluation as a decision-making tool. This would improve security for mines' manufacturers and operators. Numerous instruments, various security methods, and appropriate procedures must be applied to create a more efficient and safer mining workforce. A Proportional Risk Assessment Technique methodically identifies and analyzes risks to determine their context, influence, as well as the physical surroundings vulnerability to such risks. It aims to standardize the identification, evaluation, and regulation of workplace risks.

### 1.1 Goals of the study

This study looks at the safety and risk management practices of Chinese enterprises in Gabon's mining industry, and how these practices contribute to environmental sustainability and social responsibility. The study aims at providing the following at the end of the studies.

➢ To identify the safety and risk management practices adopted by Chinese enterprises in Gabon's mining industry.
➢ To evaluate the effectiveness of these practices in reducing accidents, injuries, and environmental impact.
➢ To examine the factors that influence the adoption and implementation of safety and risk management practices in the mining industry.
➢ To explore the relationship between safety and risk management practices and environmental sustainability and social responsibility.

### 1.2 Contribution and innovation of the study

The study identifies Gabon's mining industry's special safety problems for Chinese enterprises.

• The research evaluates Chinese mining enterprises' risk management practices.
• Comparative Analysis: The study compares Chinese enterprises' safety and risk management practices to Gabon's international and local mining industries.
• The research highlights innovative ways or technologies utilized by Chinese enterprises to improve mining safety.
• Policy suggestions: Based on the study's findings, Chinese enterprises, the Gabonese government, or other stakeholders may receive policy suggestions to improve mining safety and risk management practices.

## 2 Related works

The issues with expertise uncertainty are successfully resolved in this study, which broadens the pool of data that may be utilized to improve the accuracy of the computation findings. This could assist in simplifying the decision-making method for numerous characteristics as well as prevent the complicated method. To create and assess the evaluation model of development risk variables, researchers also use the threats of technological development concerning corporate initiatives as the study objective in this work. After all, this approach has several flaws. Quite often the outcomes are too near to affect the choices [5].

The mining sector and linked integrated technological advances have become increasingly significant due to the present growth of robots, particularly in Cyber Networks. Modern automated gigantic wagons with autonomous hauling technology that could move ore without personal interference are a result of this advancement. Autonomous hauling technology permits distant or independent control of physical processes, just like CPS. As a result, like CPS, AHS is exposed to cyber-attacks like Wi-Fi De-Auth and Global Positioning System (GPS) intrusions. Because of enhanced operational effectiveness, several mining operational processes have been improved through the usage of autonomous hauling technology. These tasks necessitate the gathering of proper information, from which exact information regarding the condition of the mines must be produced in a reliable and fast approach. As a result, having trustworthy and secure connectivity is essential for improving the safety, efficiency, and sustainability of Autonomous hauling technology mines. This essay attempts to analyze, evaluate, and emphasize the difficulties and unresolved problems related to cybersecurity, connectivity, and Autonomous hauling technology protection in the mining industry. They examine the research that addresses this goal, go over its advantages and disadvantages, and finally identify certain unresolved problems. Researchers conclude that resolving Autonomous hauling technology cybersecurity concerns can improve safety by ensuring the privacy of activities in the mining area and by enabling dependable communications. It was also discovered that modern communications technology, like 5G and Long Term Evolution (LTE), might be incorporated in Autonomous hauling technology-based mining algorithms, although much more study is required to take relevant cybersecurity risks and threats into consideration. Private (LTE is a potential replacement for conventional 802.11 technologies which might offer reliable connection and get over Wireless sensor restrictions [6].

This research examines workplace hazards and dangers associated with mineral extraction, one of the more hazardous sectors that frequently result in major work-related deaths. The research analysis is conducted underneath zinc and copper mines using the risks-based methodology that is presented. The article's findings show how constrained approaches to the fuzzy method could classify risks into various danger levels. The paper offers a conceptual development by proposing a VlseKriterijumska Optimizacija I Kompromisno Resenje (PFVIKOR) methodology based on Pythagorean fuzzy digits. Additionally, by taking into account and offering advice on possible risks associated with risk monitoring, it helps to raise the total safety requirements of deep mines. The suggested strategy would enhance the current safety risk evaluation process in deep copper and zinc mines. Estimates with specifics weren't provided for each operational region because of space restrictions [7].

Structure safety may be increased by combining this technique with previously established methodological risks. This study has the flaw of not accounting for the danger posed by the human aspect. The human component, which is frequently ignored, refers to people's talents and limits in terms of the range of their job in addition to their interactions with one another and with other people, equipment, processes, and the surroundings [8]. Researchers also established a basic accident percentage matrix that allowed us to visualize the findings of a similar evaluation of the accident percentages at the firm's manufacturing locations and identify the most important areas for enhancing occupational safety while also decreasing injury percentages. Designers have designed and built comparable accident concerns and their variations over time. It indeed needs to create software to implement the suggested algorithm for determining the prioritized regions for decreasing workplace injury rates to conduct a study [9].

Numerous aspects that necessitate empirical decision-making for the identification and prioritization of crucial aspects of accident prevention are necessary for a comprehensive enhancement in mining protection. There is an absence of proper safety programs and security teaching in the workplace atmosphere regularly. The poor state of mine protection and safety is a result of inadequate supervision of the implementation of safety activities as well as a lack of workers with high aspirations and sturdy working ethics. The employment conditions in mines continue to be challenging while there are no preventive and management processes in situ to minimize hazards and dangers. As a result, crucial concerns can be tackled in the sequence they are listed in this document in a bid to improve mining risk. Additional prioritising applications might employ the suggested analytical technique to evaluate the indicators and choose the best managerial approaches. A comparison study is constrained by the characteristic rating procedure and thus limits the ability to prioritize one variable over the others [10].

The mining industry in Gabon has experienced significant growth in recent years, with an increasing number of Chinese enterprises investing in this sector. While this investment has brought economic benefits, it has also raised concerns regarding safety and risk management practices within these operations. As such, this study aims to explore and assess the safety and risk management measures implemented by Chinese enterprises operating in Gabon's mining industry. The justification for conducting this study is rooted in the need to address critical gaps in the current literature, better understand the safety challenges faced by these enterprises, and propose effective risk management strategies to enhance workplace safety and protect both employees and the environment. The justification for conducting the study on “Safety and Risk Management of Chinese Enterprises in Gabon's Mining Industry” lies in the need to address existing knowledge gaps, enhance workplace safety, promote sustainable business practices, and facilitate international collaboration. By conducting this research and disseminating its findings, the study aims to contribute to the broader goal of creating a safer and more sustainable mining industry in Gabon while also offering valuable insights applicable to similar contexts worldwide.

## 3 Methodology

### 3.1 Data collection

Gabon is an equatorial African country located on the Atlantic Ocean coast, between Cameroon and Equatorial Guinea to the north and Congo to the south. Its size is 257,670 square kilometers, and in 2003, it was home to around 1.3 million people. Petroleum extraction, which made up 43% of the gross domestic product and nearly 77% of its total commodity exports, dominates the mining sector. In comparison, mines accounted for approximately 2 % of the total Gross Domestic Product GDP. With five additional nations, Gabon is a member of the Central African Economic and Monetary Union. Its currencies are tied to the euro (€) at a value of CFA655.957 = €1.00. The depreciation of the dollar against the euro, which resulted in the exchange ratio falling to CFA581.2 = $ 1.00 in 2003 from CFA696.99 = $ 1.00 in 2002, countered a few of Gabon's benefits from rising oil pricing in 2003. However, the launch of one minor field and better extraction efficiencies allowed productivity to supposedly grow to 268,000 barrels per day from 252,000 in 2002, but crude oil productivity has been on a downward trend since 1997. The IMF calculated that the overall investment in goods that were exported in 2003 was $2.89 billion, of which $2.23 billion was attributed to petroleum. This is an overview of the potential dangers associated with a new mining operation. To be adaptable to the vast majority of risk evaluation methods used in this industry, the researchers have developed hierarchy divisions and also methodically tracked probable variables. These tools include LTIFR, the Risk Assessment Matrix, and various KPIs. By using these tools to identify hazards, evaluate risks, and monitor performance, Chinese enterprises can prioritize safety and ensure the sustainable operation of their mining activities.

### 3.2 Proportional Risk Assessment Technique

The Proportional Risk Assessment Technique (PRAT) approach for risk evaluation is being used concurrently. A risk ratio (R) is computed for every potential threat element in Equation (1):

(1) $$R_{v}=P_{a}\times S_{f}\times F_{a}$$

where in $S_{f}$ is the degree of damage for a particular risk variable, $P_{a}$ is the likelihood that an injury type will occur, while $F_{a}$ is the frequency with which accidents of that kind take place. Following these three components, in addition to the amount R, could be stated on a range from one to 1000. The following are the levels of every element taken into account in this assessment: When determining the severity factor ($S_{f})$, took into account whether a potential risk contributed to at least one fatal occurrence each year and allocated it the highest possible level based on the typical duration for an accident to occur. Depending on the specific expert's experiences, ratings were allocated to the selected variables. Based on the information in “estimated duration for an accident to happen” that has been allocated various values to a variable $F_{a}$. d=1×48×5×16=3840hours/year Were used to determine the statistics for the columns “Events/hour” and “Estimated number for an occurrence.

**Fig. 2:**
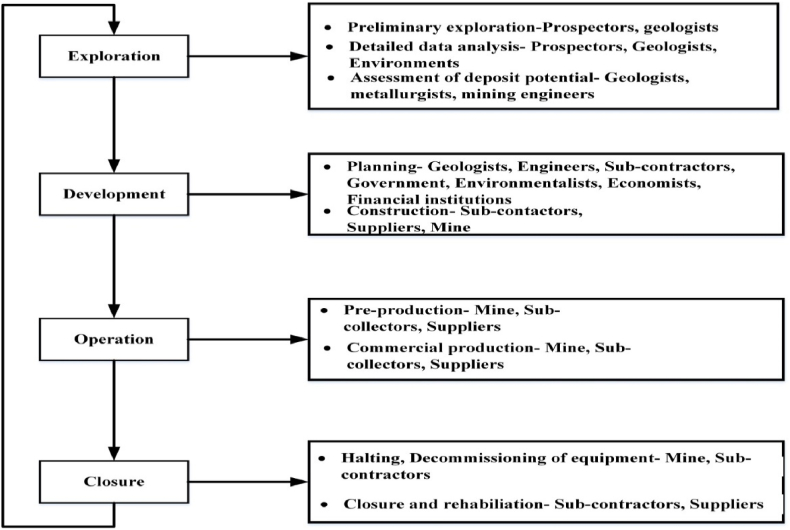
Schematic of mining life cycle process [11].

This was done by assuming that there are 48 functioning weeks every year, five business days each week, and 16 regular hours a day. It is important to note that the construction firm operates in two shifts each day, totaling 16 h. Summarize these results for each component after calculating the $R_{v}$ readings. By splitting every factor $R_{v}$ values by the sum, every factor's normalised risk level is determined. A mine venture has many hazards. Fig. 2provides a brief explanation of the four different phases that make up the entire course of a mining operation [11] (see Fig. 3).

The first 7–10 decades of exploration focused on harvestable materials. These assignments use qualitative and quantitative mineral resource assessments. Mines technologists, geologists, materials scientists, and ecologists are among the teams and experts involved in this phase. This stage involves many organizations as well. Operational teams, especially those in manufacturing and management, are the most sought-after. This phase may involve switching teams to work on new exploratory or growing projects. Like any industrial activity, mining operations include unresolved risks and uncertainty that can lead to poor decision-making [12]. All construction procedures include risks and unknown factors, including utilizing exploitation equipment. The estimation uncertainty for the number or grade of mineral resources, which exists from the discovery phase on, is the reason for the inadequate work plan. The operating stage poses risks, including Occupational Health and Safety hazards [13]. Large machinery and interconnections between electricity resources pose such risks [14]. Market volatility, competitiveness, regulatory changes, and fiscal and commercial difficulties, are other major contributors [15].n summary, many hazards vary in frequency and severity depending on their growth stage. Corporate and personnel risk threshold rates, responsibility-related issues, and preventative tactics affect how such hazards are handled [16].

### 3.3 Mining risk management

This vast occupational risk management research includes mining activities. Alarming evidence on occupational injuries, labor-related fatalities, and mining's ecological, economic, and social impacts supports studies [17]. The mining industry is one of the world's riskiest and most unpredictable sectors [18,19]. There is a dispute in the literature concerning the risk management methods that mining enterprises should apply, in contrast to many other economic sectors and regardless of the risks and uncertainties involved with mining operations. Few research studies address all mining risks. This little amount of research might be explained by a lack of exact and trustworthy information as well as knowledge to evaluate and recognize all relevant elements [20]. Risk assessment is key during mining construction. These restrictions include:(1) interaction and inclusion of team members with different opinions and cultures within a firm and communication challenges among enterprises working on the same proposal; (2) differences in risk-related laws, regulatory standards, and regulations from country to country;

(3) difficulties with squad regeneration and personnel happiness during a mining operation, which result in the loss of intellectual assets and the expertise and experience needed to meet the organization's goals. Handling these limits requires appropriate methods [21]. These procedures must be tailored to the type of mining, the resources collected, and national laws. Newer, structured, and methodical techniques that consistently resolve challenges are needed to control mining risks. Comprehensive risk management allows for prevention. A mining operation's risk assessment should cover sociological, historical, OHS, ecological, and financial hazards. This evaluation is done by an operating employee group, and excellent interaction is crucial to the reliability of risk control systems [22]. Underestimating ecological dangers, as well as financial issues, risk management aspects, bureaucratic and technical difficulties, resources, estimate inaccuracies, logistic restrictions, and industrial injuries during construction and operations, were recognized as concerns. New technologies and legal changes increase risks [23].

To avoid concerns with mine and mining instruments, apparatus adjustments, or work practice, some scientists have focused their study on technological hazards [24]. Occupational safety and health (OSH) risks include fires, employee fatigue, temperature stresses, air pollution, and disdain for safety briefings. These methodologies employ qualitative methods like Hazard analysis and FMECA; empirical methods like simulations (Arena® and Monte Carlo); or semi-quantitative methods like multiple criteria assessments [25].

This study explores enterprise risks and dangers using academic and practitioner work. Threats, mining, beneath, accessible, program management, risk assessment, the production process, fiscal, economical, operating, OHS, environmental, governmental, legal, social, and cultural elements, planning, communication, organization, and technical instruments They say a researcher's methodology was used at the end of 2010 to identify Gabon's mining sector's risks. The researchers used multiple information strategies to identify investment risks and perils. They also used 40 h of group fieldwork at operating locations, including the major mine, residue processing regions, and technical repair facilities [26].

### 3.4 Operational hazards in mineral extraction

The strategy used to create a risk portrait for a mining operation is detailed below. As far as we are aware, there is no agreement on the risk types that should be used for mineral risk assessment. The risk classifications were mostly used and modified by the researchers in the current investigation. The methodology described above was followed to construct a mining project risks portrait. To the best of my knowledge, there is no consensus as to the choice of risk categories for mining risk management. In the present study, the authors employed and adapted primarily the risk categories [27]. They were inspired by the PMBOK® Guide's risk response decomposition architecture as they organised these groups' hierarchy. Efforts were undertaken to elicit discussions for each threat area utilizing findings from instance information found in the academia as well as from the researchers proceeded. The authors also specified construction risks for every risk group to separate exogenous causes [28].

#### 3.4.1 Operational risks

Risks classified as operating hazards are grouped as organizational hazards, design threats, manufacturing threats, and technical risks. It may also evaluate societal concerns connected to the business. Risk assessments, management risks, design risks, manufacturing risks, and technical risks are grouped as functional hazards. It could also involve societal hazards connected to the enterprise. Put power source and equipment risks of damage and death under operations concerns rather than safety concerns [29]. Design and mining operating requirements are highlighted as operational aspects that can cause interruptions. It emphasizes technological hazards at mining sites and in tunnel buildings. Deal with component construction hazards and use stochastic structure analysis to choose component proportions [30]. Management concerns may affect a company's workplace. Technological risk management promotes corporate efficiency by ensuring reliable mining operations. Skilled labor is essential due to its importance. Qualified mining personnel is few worldwide. Regional and international issues complicate the situation. Retaining personnel is becoming a major risk [31].

#### 3.4.2 Financial and economic risks

Mining businesses must continually adjust their expenditures, collaborate with other businesses, and prioritize contracting work if they want to prevent huge costs. Mining development assessment studies use several financial and economic aspects to determine venture viability. This research's dependability is contingent on the accessibility and precision of technical information [32]. For evaluating concerns and uncertainty associated with fiscal, economic, and technological factors, several scholars have presented frameworks. Due to a mining development's intricacy, these individual research shows their limitations when it comes to covering all aspects of the project. Additionally, mining firms are quite susceptible to changes in the cost of materials. These rates are regulated on several marketplaces and are based on demand around the world. These factors make dependence upon currency rates and market movements extremely significant. Many times, these oscillations are considered in feasibility analysis for exploration activities. Market information is unpredictable and is based on compiled statistics. The viability analyses of exploration activities are made more unclear by these limits. Such analyses are also susceptible to data on integrity and competitiveness being available. Mining should also think about the substantial hazard of exposure to funding. The enterprises' ability to access funding enables them to consistently upgrade and replace tools and means, develop new operations, and discover different resources.

#### 3.4.3 Political and constitutional concerns

Regulatory and legal reforms might occasionally introduce new measures that could increase exploitation expenses and make managing a business more difficult. For instance, changes to Polish environmental protection rules in the 1980s resulted in disproportionate waste treatment and retention expenditures that copper companies were not prepared for. These inevitable price rises have the potential to cause deficits and the closure of the mining industry. One such is the recent legislation that the administration of Gabon enacted. When this rule is passed, mining corporations will suffer major financial repercussions, including reduced subsidies and tax holidays [33].

#### 3.4.4 Ecological concerns

Ecological concerns have been the subject of numerous investigations, whether they occur during mining operations or following an early or scheduled mining abandonment. It is significant to note that during organizational stages such as water resource polluted air, loud noises, environmental contamination, debris, radioactivity, and so forth, or even during closing stages such as lengthy consequences of radiation industrial chemicals, iron ore waste material, and others., investigators are preoccupied with ecological concerns. Even among the more highly controlled nations in this area, the harmful effects of mineral extraction on the neighborhood and the environment are evident regularly. Most job involves mining, refining, and using mineral ore deposits, which causes ecological threats. Miners employ procedures for ore extraction that utilize a variety of organic chemicals during the extraction stage. The most recent technology allows for the recovery of the greatest quantity of these goods as well as the discharge of sterilized waste. Because miners all over the globe lack access to the latest machinery generations, the issue of toxic contamination caused by earlier techniques is still being debated [34].

#### 3.4.5 Occupational health and safety risks

In many affluent nations, such as the US and Canada, mining injuries and diseases have decreased [11]. The mining business and regulators' safety efforts caused this decline. Occupational Health and Safety risks include mine machinery, natural calamities, mining corporations, working conditions, and falling rocks or galleries [35]. OHS examines skill gap issues. Experts suggested integrating and developing new hires and improving working conditions. OHS risks produce many different hazards. Hazards include mechanical (machinery, automobiles, washing, and servicing), electronic (electric power source materials and electronic devices), ecologic (heat generation, moisture, debris, loud sounds, and acoustic noise), social and human (dangerous activities, exhaustion, and expertise), and management styles [36]. Operator error causes mining companies diseases, injuries, and deaths. Human errors are hard to detect and quantify using risk evaluation methods. Personal failure is linked to unsafe behavior, a lack of knowledge, ability, or competency, and hazardous perceptions [37]. These elements—man-machine interactions, workplace culture, procedures and techniques, knowledge and training, team leadership, security systems, administrative staff, internal organization, and safety protocols—show how human mistakes affect other criteria. These factors affect how employees and supervisors view and take risks.

#### 3.4.6 Safety management systems

A crucial necessity for mine worker health and well-being is the creation and deployment of a safety management system (SMS). The safety management system's main goal is to outline the specific parts of the mining works activities that have an impact on the security of employees as well as other site visitors effectively and systematically. A safety management system will record. This would describe the mining operator's goals according to how he or she will handle safety results. Considering the nature and intricacy of the mining companies and the hazards connected with those activities, this should contain an adequate degree of information on how hazards to security and health would be controlled. A tiny quarry or emerald mine won't have to report its mining activities to the same extent as a big, complicated mining project like Olympic Dam, for instance. The safety management system needs to be recorded. Lost Time Injury Frequency Rate (LTIFR) metric is a widely used measure in the mining industry to assess safety performance. It is calculated by dividing the number of lost-time injuries by the total number of hours worked and multiplying the result by one million. Lost Time Injury Frequency Rate (LTIFR):

The formula for LTIFR is:

LTIFR = (Number of lost-time injuries/Total hours worked) x 1,000,000.

Chinese mining company operating in Gabon reports six lost-time injuries in a year, and their employees worked a total of 150,000 h during the same period, the LTIFR for this company as seen in the Equations (2), (3) respectivey:

(2) LTIFR=(6/150,000)x1,000,000=40

(3) LTIFR=(4/100,000)x1,000,000=40

Table of LTIFR by the company - The below shows the LTIFR for different Chinese mining companies operating in Gabon, allowing for comparisons between companies (see Table 1).

**Table 1:** Comparisons of different Chinese mining companies operating in Gabon.

Company Name	Number of lost-time injuries Total hours worked		LTIFR
Company A	4	100,000	40
Company B	2	50,000	20
Company C	8	200,000	80

**Graph 1:**
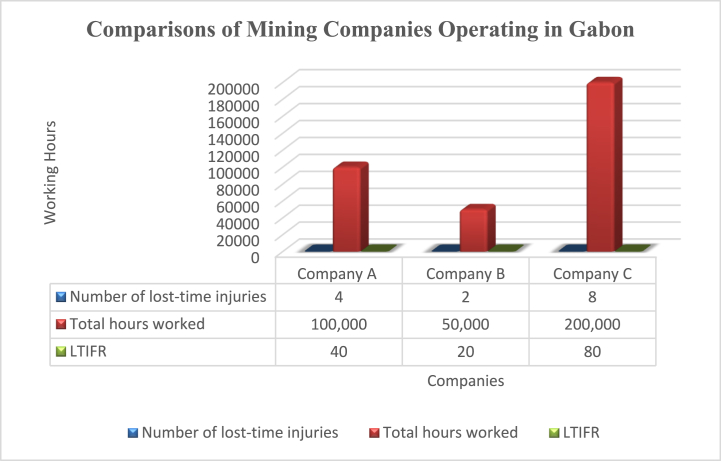
Comparisons of different Chinese mining companies operating in Gabon.

This means that the company experienced 40 lost-time injuries per million hours worked, which can be compared to industry benchmarks to evaluate their safety performance as shown in Graph 1 above. To manage risk effectively, Chinese enterprises in Gabon's mining industry must also use analytical tools such as risk assessments and monitoring. Risk assessments involve identifying and evaluating potential hazards and their associated risks, and implementing measures to mitigate those risks. This can be done using key performance indicators (KPIs) such as the number of incidents, near misses, and safety audits conducted.

#### 3.4.7 Risk Assessment Matrix

The Risk Assessment Matrix is a tool used to evaluate the likelihood and severity of potential hazards and associated risks. It involves assigning scores to each hazard based on these factors and then plotting the scores on a matrix to determine the level of risk. The formula for calculating the risk score is shown in Equation (4):

(4) RiskScore=LikelihoodxSeverity

If the hazard has a likelihood score of 4 (out of 5) and a severity score of 3 (out of 5), the risk score is shown in Equation (5):

(5) RiskScore=4x3=12

This matrix is used to assess and prioritize risks based on their likelihood and severity, with risks ranked from highest to lowest priority (see Table 2).

**Table 2:** Assessment and prioritize risks based on their likelihood and severity ranked.

Likelihood/Severity	High(4)	Medium(3)	Low(2)	VeryLow(1)
High(4)	16	12	8	4
Medium(3)	12	9	6	3
Low(2)	8	6	4	2
Very Low(1)	4	3	2	1

**Graph 2:**
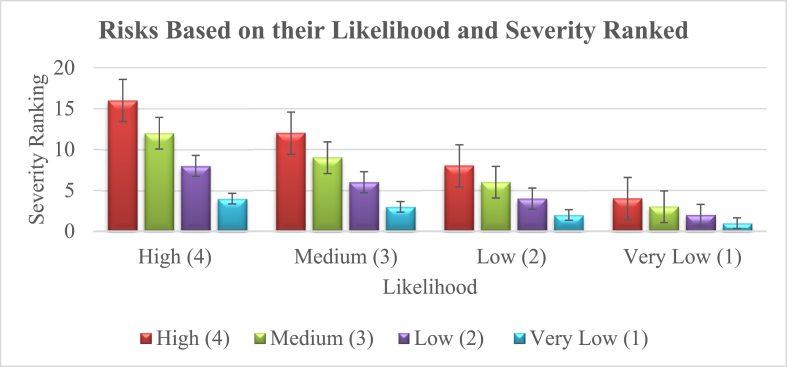
Assessment and prioritize risks based on their likelihood and severity ranked. The table above it can be noted the rank of the risks and their likelihood to disrupt the activities of the mines. From its severity and its possibility of happening. The higher the risk the more severe its impact is on the operations of the mines and vice versa. It is emphasized that when determining the key risks to mines, assessment, and prioritization are crucial. According to Graph 2, each element's (Likelihood and Severity) assigned number denotes a degree of relevance, such as high, medium, low, or very low. It's crucial to remember that a risk matrix does not aim to provide a precise, “quantitative” calculation of risk.

## 4 Result and discussion

**Graph 3:**
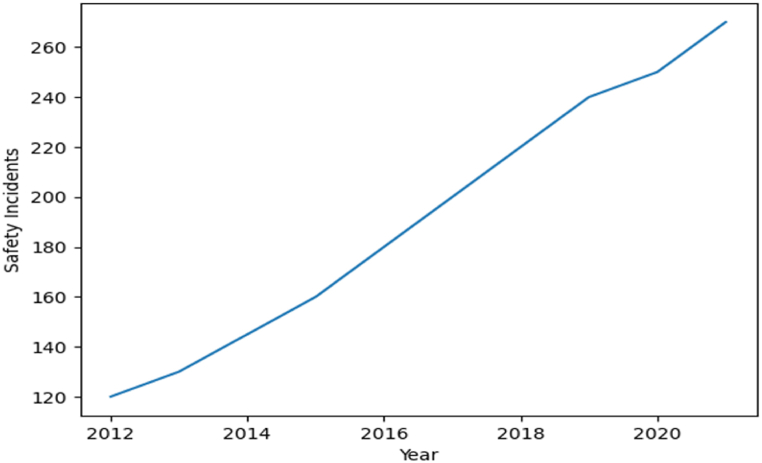
Safety Incidents over Time of Chinese mining company in Gabon (2012–2021).

The management of all mining risks needs to be addressed to address any potential issues that may arise throughout the entirety of the mission. It does not currently have a structure, nor does it have a systematic approach. Researchers and subject matter experts have successfully managed often-targeted problem areas within severely tight timelines by employing a variety of strategies that have been borrowed from a wide range of organizations. The investigated piece successfully controls several risks as a result of the probable issue that was brought up as well as the authors' area of expertise. The vast bulk of work has been put into attempting to find solutions to problems that have surfaced during the construction and operation stages of mining projects [2,29]. The presence of several limits and risks connected to extensive outdoor activities justifies the focus on these phases of development. Expertise in information collection is difficult to find, yet it is necessary for the operation of risk assessment systems. The recognition of all consequences associated with the same proposal is hampered by several variables, such as time restraints for risk management, a lack of understanding and specialist information regarding the vast majority of mining operations, the complexity of assembling interdisciplinary risk management squads, a heavy reliance on subcontractors and the organizational issues that result from this, and issues with data accessibility and sharing regarding the majority of current consequences. It is constrained to introducing prospective risks and latent events, and it has difficulty determining how different threats reinforce one another. Despite the presence of a qualified multidisciplinary team and the availability of resources, mining risk assessment is made more difficult due to the complexity of exploration activities, the diversity of dangers, the interrelationships within organizations, and the dependence on several external variables that have a significant impact Graph 3.

This graph could show the number of safety incidents reported by a Chinese mining company in Gabon over a specific period, such as a year. The graphs can help to visualize and analyze data related to safety and risk management in Gabon's mining industry, allowing Chinese enterprises to make data-driven decisions to improve their operations. This was done using the Risk Assessment Matrix, which ranks hazards based on their likelihood and severity. The risks identified throughout mining sites are of numerous kinds, with a range of potential effects that could jeopardize the company's operations as a whole. The sophistication of determining the dangers associated with a single mining procedure and the amount of employment necessary to incorporate these constructively may be demonstrated to readers by the monetary consequences, uncertainties of the cost of metal, risks associated with mining asset estimates, strategic risk, medical problems, operating parameters, and ecologic areas of concern. This campaign demonstrates how crucial it is to list all current risks identified along with any potential effects to fully understand how they arise and the harm they can do to both the project and the company.

## 5 Conclusion

Mining endeavors are inherently unpredictable and fraught with a significant degree of risk. Because mining alters ecosystems, mining companies must deal with a variety of hazards during their operations. The work that investigators do helps handle a wide variety of hazards because of the issues that they have uncovered and the areas of competency in which they are skilled. The management of all mining hazards is not methodical or complete, so it needs to be improved to adequately address the concerns that can arise as a project proceeds forward. This is because it is currently in need of improvement. To accomplish the task at hand, which was to produce a first picture of the hazards that have been recognized in mining, the authors relied heavily on the findings that came from the many studies that have been carried out in recent years. The findings of their work identifying risks in Gabon's mining sector were used to create this graphic. The image that has been drawn lends credence to the existence of several important construction risks, some of which include operational, financial, economic, legal, political, environmental, and occupational health and safety hazards. The current research reveals the potential for identifying numerous classes of known risks as well as unknowns that are not usually taken into consideration as a whole in the management of the risks associated with mining activities. To highlight their effects and frequency concerning the various stages of a mining operation, the detected hazards are grouped in hierarchical frameworks. This study also exhibits impacts among many identified hazards and their potential growth concerning the stages of a mining operation. To keep mines and workers safe, it is vital to conduct an assessment of the risks to safety and the environment. It is necessary to evaluate the risk posed by various mining activities to prevent, eliminate, and reduce the risk. Both qualitative and quantitative methods could be used to evaluate the level of risk. In this particular piece of work, the risk was evaluated using the PRAT (Proportional Risk Assessment Technique) method. It has the potential to become a method that can investigate and comprehend overall risk more effectively, as well as assist in avoiding and managing risks. a deeper comprehension of the safety and risk management practices implemented by Chinese companies operating in Gabon's mining industry. determination of the factors that affect the acceptance and application of these practices Evaluation of the effectiveness of these practices in lowering the number of accidents, injuries, and negative effects on the environment Recommendations for enhancing mining industry practices related to safety and risk management, as well as recommendations for fostering environmental sustainability and social responsibility. Monitoring needs to be done regularly so that the effectiveness of risk management strategies may be evaluated and adjusted as required. To generate key performance indicators (KPIs), data must be collected consistently and then compared to benchmarks or targets to see where there is room for improvement.

In conclusion, while the current study offers a valuable initial picture of the hazards in Gabon's mining industry, it also highlights the need for further research to address the limitations and explore additional dimensions of safety and risk management. Further research in the suggested directions can contribute to a more comprehensive understanding of safety challenges in the mining sector and lead to more effective risk management strategies that prioritize the well-being of workers, communities, and the environment.

## Funding

This study was supported by the 10.13039/501100001809National Natural Science Foundation of China under grant No. 71971003), the Major of 10.13039/501100012456National Social Science Foundation of China (No.22ZDA112).

## Data availability statement

Yes, request to this email: inespamelanguembi@gmail.com.

## CRediT authorship contribution statement

**Ines Pamela Nguembi:** Conceptualization, Data curation, Methodology. **Li Yang:** Formal analysis, Funding acquisition, Supervision, Writing – review & editing. **Vincentia Serwah Appiah:** Formal analysis, Investigation, Methodology, Software.

## Declaration of competing interest

The authors declare that they have no known competing financial interests or personal relationships that could have appeared to influence the work reported in this paper.

## References

[1] Brady T., Iannacchione A.T., Varley F. (2008).

[2] Diogo M.P., Van Laak D. (2016).

[3] Southall R., Melber H. (2009).

[4] Morgan P. (2019). Can China's economic statecraft win soft power in Africa? Unpacking trade, investment and aid. J. Chin. Polit. Sci..

[5] Zhu D., Wang X. (2016). Chinese Control and Decision Conference (CCDC).

[6] Gaber T., El Jazouli Y., Eldesouky E., Ali A. (Jun. 2021). Autonomous haulage systems in the mining industry: cybersecurity, communication and safety issues and challenges. Electronics.

[7] Gul M., Ak M.F., Guneri A.F. (Jun. 2019). Pythagorean fuzzy VIKOR-based approach for safety risk assessment in mine industry. J. Saf. Res..

[8] Kirin S. (Aug. 2021). Human factor risk management procedures applied in the case of open pit mine. Eng. Fail. Anal..

[9] Gendler S., Prokhorova E. (Mar. 2021). Risk-based methodology for determining priority directions for improving occupational safety in the mining industry of the arctic zone. Resources.

[10] Jiskani I.M., Han S., Rehman A.U., Shahani N.M., Tariq M., Brohi M.A. (Aug. 2021). An integrated entropy weight and grey clustering method–based evaluation to improve safety in mines. Min. Metall. Explor..

[11] Badri A., Nadeau S., Gbodossou A. (2012). A mining project is a field of risks: a systematic and preliminary portrait of mining risks. Int. J. Saf. Secure. Eng..

[12] Chinbat U., Takakuwa S. (Dec. 2009). Proceedings of the 2009 Winter Simulation Conference.

[13] Kumar P., Kumar A. (2016). Methods for risk management of mining excavator through FMEA and FMECA. Int. J. Eng. Sci..

[14] Ghosh A.K. (2010). Risk assessment of occupational injuries in underground coal mines. J Mines Met Fuels.

[15] Abdel Sabour S., Wood G. (2009). Modelling financial risk in open pit mine projects: implications for strategic decision-making. J. South. Afr. Inst. Min. Metall..

[16] Galvin J. (2006).

[17] Fourie A., Brent A.C. (Jan. 2006). A project-based mine closure model (MCM) for sustainable asset life cycle management. J. Clean. Prod..

[18] Luo H., Liu B. (2010). International Conference on Management and Service Science.

[19] Janjuhah H.T., Ishfaque M., Mehmood M.I., Kontakiotis G., Shahzad S.M., Zarkogiannis S.D. (2021). Integrated underground mining hazard assessment, management, environmental monitoring, and policy control in Pakistan. Sustainability.

[20] Atkinson T., Allington R., Cobb A. (1996). Risk management for mining projects. Min. Technol..

[21] Schafrik S., Kazakidis V. (2011). Due diligence in mine feasibility studies for the assessment of social risk. Int. J. Min. Reclamat. Environ..

[22] Evans R., Brereton D., Joy J. (2007). Risk assessment as a tool to explore sustainable development issues: lessons from the Australian coal industry. Int. J. Risk Assess. Manag..

[23] de los Reyes J.A. (2022). Re-making Pascua Lama: corporate financialization and the production of extractive space. J. Peasant Stud..

[24] Gunaratnam K., Abdullah A., Bakar R.A., Hujainah F. (2022). Hazard analysis techniques, methods and approaches: a review. Int. J. Adv. Res. Eng. Innov..

[25] Li Q., Wang Z., Lu L., Ma Q. (2021). Construction risk evaluation of poor geological channels based on cloud model-improved AHP–matter–element theory. Sustainability.

[26] Brisbois B. (2021). Storylines of research on resource extraction and health in Canada: a modified metanarrative synthesis. Soc. Sci. Med..

[27] Stolar A., Friedl A. (2021). Process safety for sustainable applications. Int. J. Reliab. Qual. Saf. Eng..

[28] Varajão J., Marques R.P., Trigo A. (2022). Project management processes–impact on the success of information systems projects. Informatica.

[29] Grusenmeyer C. (2007).

[30] Najafi M., Jalali S., Bafghi A.Y., Sereshki F. (2011). Prediction of the confidence interval for stability analysis of chain pillars in coal mines. Saf. Sci..

[31] Louit D., Pascual R., Banjevic D., Jardine A.K. (2011). Optimization models for critical spare parts inventories—a reliability approach. J. Oper. Res. Soc..

[32] Bascetin A., Tuylu S., Nieto A. (2011). Influence of the ore block model estimation on the determination of the mining cutoff grade policy for sustainable mine production. Environ. Earth Sci..

[33] Maurice P. (2004).

[34] Masto R.E. (2011). Impacts of open cast coal mine and mine fire on the trace elements' content of the surrounding soil vis-a-vis human health risk. Toxicol. Environ. Chem..

[35] Badri A., Nadeau S., Gbodossou A. (2012). A mining project is a field of risks: a systematic and preliminary portrait of mining risks. Int. J. Saf. Secure. Eng..

[36] McBride D.I. (2004). Noise-induced hearing loss and hearing conservation in mining. Occup. Med..

[37] Patterson J.M., Shappell S.A. (2010). Operator error and system deficiencies: analysis of 508 mining incidents and accidents from Queensland, Australia using HFACS. Accid. Anal. Prev..

